# Prospective Association of Air-Purifier Usage during Pregnancy with Infant Neurodevelopment: A Nationwide Longitudinal Study—Japan Environment and Children’s Study (JECS)

**DOI:** 10.3390/jcm9061924

**Published:** 2020-06-19

**Authors:** Kenta Matsumura, Kei Hamazaki, Akiko Tsuchida, Hidekuni Inadera, The Japan Environment and Children’s Study (JECS) Group

**Affiliations:** 1 Toyama Regional Center for Japan Environment and Children’s Study, University of Toyama, 2630 Sugitani, Toyama, Toyama 930-0194, Japan; keihama@med.u-toyama.ac.jp (K.H.); aktsuchi@med.u-toyama.ac.jp (A.T.); inadera@med.u-toyama.ac.jp (H.I.); 2 Department of Public Health, Faculty of Medicine, University of Toyama, 2630 Sugitani, Toyama, Toyama 930-0194, Japan; 3 JECS Programme Office, National Institute for Environmental Studies, 16-2 Onogawa, Tsukuba, Ibaraki 305-8506, Japan; jecscore@nies.go.jp

**Keywords:** air purifier, air cleaner, developmental delay, dwelling environment, indoor environment, birth cohort

## Abstract

Fetal exposure to particulate matter (PM) is associated with infant developmental delay likely via neuroinflammation and prefrontal cortex lesions; however, whether air-purifier usage, which can reduce indoor PM levels, is related to infant developmental delay remains unknown. We therefore examined the prospective relationship between air-purifier usage during pregnancy and infant developmental delay by analyzing 82,441 mother–infant pairs using a simple yes/no questionnaire. Developmental delays at 6 and 12 months were assessed in five areas using the Ages and Stages Questionnaire, Third Edition. A generalized linear mixed model analysis was used to derive adjusted odds ratios (AORs) and 95% confidence intervals (95% CIs) while controlling for 20 covariates. The analysis revealed that air-purifier usage was associated with developmental delays in fine motor (AOR: 0.91, 95% CI: 0.83–0.99) and problem solving (AOR: 0.83, 95% CI: 0.77–0.90) at 6 months and in communication (AOR: 0.86, 95% CI: 0.79–0.93), fine motor (AOR: 0.87, 95% CI: 0.82–0.92), problem solving (AOR: 0.83, 95% CI: 0.77–0.88), and personal–social (AOR: 0.79, 95% CI: 0.72–0.86) at 12 months. In conclusion, a negative association exists between air-purifier usage during pregnancy and infant neurodevelopmental delay that strengthens with time. Our results outline the potential role of air purifiers in inhibiting infant neurodevelopmental delay.

## 1. Introduction

Pollution is the leading environmental cause of disabilities and early death [1]. Among the different types of pollution, air pollution accounts for the most disability-adjusted life years (DALYs) in the first stage of human life [2]. As such, it is a threat to children’s lives [3]. The causative agents of air pollution include particulate matter (PM), such as organic or elemental carbon, polycyclic aromatic hydrocarbons (PAHs), and metals, as well as carbon monoxide, ozone, nitrogen dioxide, and sulfur dioxide [4,5]. Indoor air pollution is considered more harmful than outdoor pollution, as people tend to spend more time indoors [6].

Exposure to PM during pregnancy has been shown to exert adverse effects on offspring. For example, Guxens et al. [7] revealed that high levels of PM around mothers’ homes during pregnancy are related to alterations in brain morphology and reduced cognitive function in school-aged children. In another example, Perera et al. [8] revealed that high levels of exposure to PAHs attached to airborne vapors and PMs in a mother’s house during pregnancy decrease a child’s full-scale and verbal IQs at the age of 5 years by 4.3 and 4.7, respectively. In fact, review articles [4,9] regarding air pollution and child neurodevelopment have reported that air pollution during pregnancy contributes to impaired cognitive and psychomotor development in children.

Although the detailed mechanisms underlying the relationship between high fetal exposure to PM and impaired child neurodevelopment remain unknown, one possible pathway possibly involved is neuroinflammation. The basis of this hypothesis is the fact that pregnancy and the first year of life are critical periods of vulnerability for human neural development [10,11]. Thus, PMs with an aerodynamic diameter <2.5 µm (PM2.5) can easily pass through the immature and thin blood–brain barrier and facilitate innate neuroinflammation, which leads to cell loss, neurodegeneration, or neuronal damage in the central nervous system [5]. In support of this view, children exposed to high PM levels show high rates of lesions in the prefrontal cortex, which is responsible for cognitive abilities [12]. In addition, a recent study showed that magnetite nanoparticles can also exert harmful effects on child neurodevelopment via increased oxidative stress and/or free radicals [13].

One countermeasure to air pollution may be the use of air purifiers at home. Present-day air purifiers are often equipped with high-efficiency particulate air (HEPA) filters, which can catch PM2.5 with over 99.97% efficacy at the rated airflow. Previous studies [14,15] have also revealed that air purifiers, even those with non-HEPA filters, can drastically reduce indoor PM1–PM50 concentrations. Since the use of air purifiers weakens allergic symptoms by reducing airborne PM2.5 levels [16], these devices can theoretically also prevent impaired neurodevelopment in infants via the elimination of such particles. Nevertheless, to date, no data are available regarding the effect of air-purifier usage on child neurodevelopment.

Therefore, in this study, we examined the prospective relationship between the use of air purifiers during the fetal period and subsequent neurodevelopment. We assessed five major developmental areas across time using a large dataset from the Japan Environment and Children’s Study (JECS), a nationwide birth cohort with *n* > 80,000 records, while controlling for possible confounders such as basic characteristics and dwelling environment. We hypothesized that the use of air purifiers is related to intact neurodevelopment.

## 2. Materials and Methods

### 2.1. Study Design and Participants

The detailed design and baseline characteristics of the JECS have been published elsewhere [17,18,19]. Briefly, the JECS is a nationwide, government-funded birth cohort study of various environmental factors and children’s health and development. In this study, 103,062 pregnancies, typically during the first trimester, were registered via face-to-face recruitment at public agencies across 19 regions, including both rural and urban locations throughout Japan (from the subpolar northern island, Hokkaido, to the southern island, Okinawa), between January 2011 and March 2014. The sample size was determined in advance to have adequate statistical power to examine conditions with ≤1% prevalence. The eligibility criteria for participants (expectant mothers) were as follows: (1) residence in the study areas at the time of recruitment and expected to reside continuously in Japan for the foreseeable future; (2) expected delivery date between 1 August 2011, and mid-2014; and (3) ability to comprehend the Japanese language and complete the self-administered questionnaire. Those residing outside the study areas, even if they visited the cooperating health-care providers within the study areas, were excluded from the study.

The JECS protocol was approved by the Ministry of the Environment Institutional Review Board for Epidemiological Studies (100910001) and by the Ethics Committees of all participating institutions. This study was also approved by the Ethics Committee, University of Toyama (R2018023) on 12 June 2018. The JECS was conducted in accordance with the Declaration of Helsinki and other nationally valid regulations, and written informed consent was obtained from all participants.

Follow-ups were conducted primarily via mailed letters and scheduled in-hospital check-ups until one month postpartum and via mailed letters at 6 and 12 months. Data were acquired using self-administered questionnaires or transcriptions of medical records recorded by physicians, midwives/nurses, and/or research coordinators. The dataset used in this study, jecs-an-20180131, was released in March 2018 and updated in December 2018. The dataset includes measurements for the first and second/third trimesters as well as for 0, 1, 6, and 12 months postpartum.

**Figure 1:**
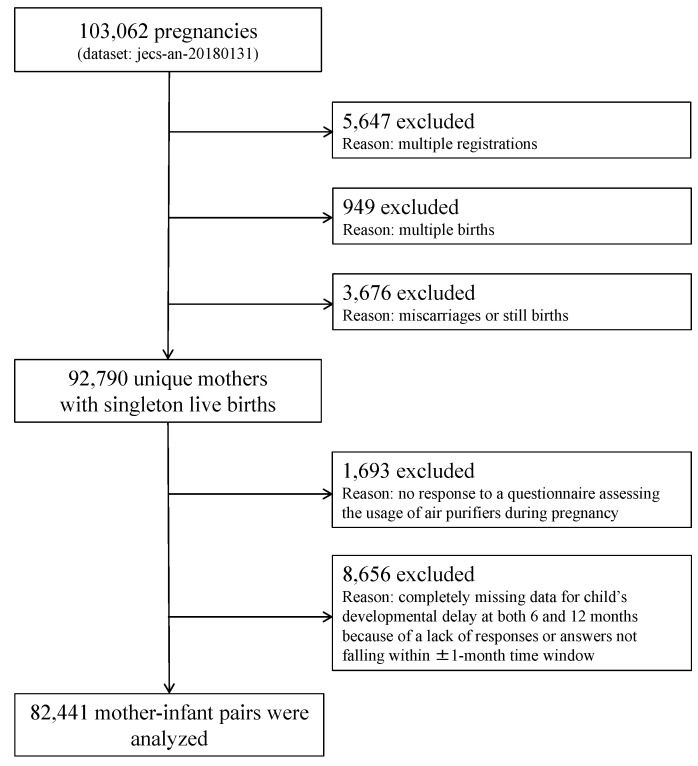
Study flow chart.

Among the 103,062 participants in this dataset, 5647 were excluded due to multiple registrations, 949 due to multiple births, and 3676 following miscarriages or stillbirths. Among the remaining 92,790 unique pregnancies with singleton live births, 1693 were further excluded due to no response to the questionnaire requesting information on demographics, psycho-social status, residence type, and dwelling environment characteristics, including the usage of air purifiers, during pregnancy. A further 8656 pregnancies were further excluded due to a lack of responses to the posted Ages & Stages Questionnaire, Third Edition (ASQ-3) [20], at both 6 and 12 months postpartum. Thus, data from 82,441 mother–child pairs were analyzed (Figure 1).

### 2.2. Measures

#### 2.2.1. Exposure

The use of air purifiers during the previous year was assessed using simple yes/no questions. The data were collected during the second/third trimester.

#### 2.2.2. Outcomes

Children’s neurodevelopment at 6 and 12 months after birth was assessed using the ASQ-3, an age-specific, structured, parent-completed child monitoring system [20]. The ASQ-3 is a set of well-validated questionnaires that has been recommended by the United Nations Children’s Fund to verify whether children have normal neurological development [21]. The ASQ-3 assesses the following five areas of development: (1) communication: language skills, such as babbling, vocalizing, listening, and understanding; (2) gross motor: arm, body, and leg movements to move and play; (3) fine motor: hand and finger movements; (4) problem solving: problem-solving skills, learning, and playing with toys; and (5) personal–social: self-help skills, solitary social play, and play with toys and others. Each area consists of six developmental items. Each parental response—yes, sometimes, and not yet—yields 10, 5, and 0 points, respectively, and the total score for each area ranges from 0 to 60 points. Screen-positive cases for each area are defined as those with scores on or below the respective threshold values, which are set at −2 standard deviations from the mean (i.e., z-score ≤ −2) to yield a sensitivity of 85–92%, a specificity of 78–92%, and a positive predictive value of 32–64% [20]. Taking early delivery into account, if a parent’s completion date at 6 and 12 months was not within ±1 month from the estimated delivery date, the data were treated as missing values in accordance with the scoring guidelines.

#### 2.2.3. Covariates

We used the following variables as potential confounders for mothers: maternal age (<25, 25–<30, 30–<35, or ≥35 years), body mass index (BMI) (<18.5, 18.5–<25, or ≥25 kg/m^2^), parity (primipara or multipara), smoking status (never, former, or current), passive smoking status (almost never or 1, 2–3, 4–6, or 7 days/week), alcohol intake (never, former, or current), number of hours spent outdoors (<1, 1–<2, 2–<3, or ≥3 h), physical activity (yes or no), folic acid intake (≤151, 152–202, 203–256, 257–337, or ≥338 µg) [22], marital status (married, single, divorced, or widowed), highest educational level (≤12, 12–<16, or ≥16 years), employment status (yes or no), and annual household income (<4, 4–<6, or ≥6 million JPY). As potential confounders for dwelling environment, we used type of residence (wooden detached house, steel-frame detached house, wooden multiple dwelling house/apartment, steel-frame multiple dwelling house/apartment, or other), high-rise living (living ≥6th floor or not) [23], number of rooms in the house/apartment (≤2, 3, 4, 5, or ≥6 *n*), living-room flooring material (tatami [Japanese straw floor covering], carpet on tatami, wooden flooring/tiles, carpet on wooden flooring/tiles, or other), age of house/apartment building (<1, 1–<3, 3–<5, 5–<10, 10–<20, or ≥20 years or unknown), house renovation/interior completion after becoming pregnant (yes or no), and number of years living in the current place of residence (<1, 1–<3, 3–<5, 5–<10, 10–<20, or ≥20 years). These covariates include standard variables for socioeconomic status and dwelling environment. In addition, we selected several variables in terms of the possibility of impact on exposure and/or outcome. Potential mediators were not used as covariates. The categorization of these variables was conducted according to usual medical practice or common practice in Japan and/or by referring to our previous studies (e.g., [24,25]). All continuous variables were categorized in advance if complex relationships existed.

### 2.3. Statistical Analysis

Separate generalized linear mixed models, with 19 regions set as a random effect and logit as a link function, were used to calculate odds ratios (ORs) and their 95% confidence intervals (CIs). Nonuse of air purifiers was used as the reference. Outcome variables were screen-positive cases in the five developmental areas: (a) communication; (b) general motor; (c) fine motor; (d) problem solving; and (e) social-personal (in the ASQ-3); as well as (f) the total number of screen-positive cases over the five areas at 6 and 12 months. The exposure variable was the yes/no answer to the question regarding the use of air purifiers.

The forced entry method was used to include covariates in the multivariate analysis. In the crude model, only the region was set as a random effect. In model 1, the regression models were adjusted for maternal age, parity, smoking status, passive smoking status, folic acid intake, marital status, highest educational level, annual household income, type of residence, number of rooms in the house/apartment, materials covering the floor of the living room, and age of house/apartment building, with the region set as a random effect. In model 2, the regression models were adjusted for the covariates used in model 1: BMI, alcohol intake, number of hours spent outdoors, physical activity, employment status, high-rise living, house renovation/interior completion after becoming pregnant, and number of years living in the current place of residence.

SAS 9.4 (SAS Institute Inc., Cary, NC, USA) and R 4.0.0 were used for all statistical analyses.

### 2.4. Missing Data

The effective response rates at 6 and 12 months postpartum were 88.01% (*n* = 81,669) and 85.60% (*n* = 79,427), respectively, while 0.83% (*n* = 772) of the mothers did not respond at 6 months but responded at 12 months. Of the 82,441 mother–infant pairs included in the study, the missing-data rate was ≤1% for all covariates except for parity (2.51%; *n* = 2073), number of years living in the current place of residence (3.13%; *n* = 2580), physical activity (3.33%; *n* = 2749), average number of hours spent outdoors (4.04%; *n* = 3329), and annual household income (6.92%; *n* = 5704). For exposure measures, the item regarding the use of air purifiers had a missing-data rate of <0.37% (*n* = 298). Each score of the five areas of the ASQ-3 had a <5.99% (max *n* = 4931) missing-data rate at 6 months and <10.57% (max *n* = 8712) at 12 months. A total of 18,985 (23.03%) and 21,908 (26.57%) mother–infant pairs had at least one missing value at 6 and 12 months, respectively.

We conducted imputation using chained equations [26] to obtain 24 imputed datasets. All data were imputed simultaneously, regardless of the measured time points. When conducting multiple imputations, auxiliary variables that were likely associated with the analyzed variables were also included to avoid violating the missing-at-random assumption. The estimates from each dataset after multiple imputation were combined using Rubin’s rule [27].

### 2.5. Sensitivity Analysis

The resulting OR patterns from the complete case datasets (*n* = 60,660–63,790) were compared with those from the multiply imputed datasets (*n* = 82,441 each) to assess the differences between the strategies for addressing missing values.

Instead of using the cases, the raw scores for each area (ranging from 0 to 60; the higher, the better) were analyzed using the same setting except for using a Gaussian distribution.

## 3. Results

### 3.1. Backgrounds

The characteristics of mothers and dwelling environments together with data regarding the use of air purifiers during pregnancy are presented in Table 1. Higher usage of air purifiers was generally associated with high education levels, high income, less smoking, married status, parity, and living in a newer house.

### 3.2. Main Results

Multicollinearity was not detected among all covariates; that is, all generalized variance inflation factors were below 2.73.

The prevalence, cases, crude ORs, and adjusted ORs (models 1 and 2) for developmental delay in each developmental area at 6 and 12 months according to the use or nonuse of air purifiers are presented in Table 2.

The mean and SD values of the total numbers of screen-positive cases over the five developmental areas and their crude ORs and adjusted ORs (models 1 and 2) according to the use or nonuse of air purifiers are presented in Table 3.

**Figure 2:**
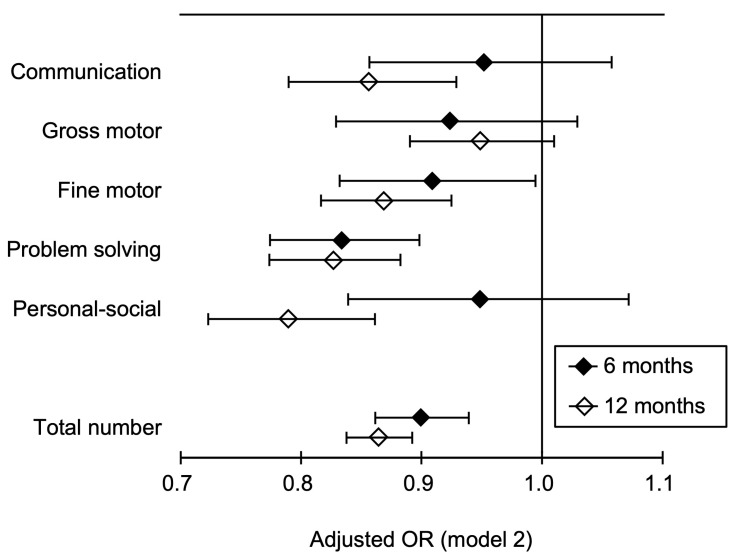
Adjusted ORs (model 2) and 95% confidence intervals (bars) of air-purifier usage for screen-positive cases of developmental delay assessed using the ASQ-3 with air-purifier nonuse set as a reference. This graph was drawn using the data shown in Table 2 and Table 3. OR, odds ratio; ASQ-3, Ages & Stages Questionnaire, Third Edition; Total number, total number of screen-positive cases over five developmental areas in the ASQ-3. Model 2 = full model adjusted for maternal age, body mass index, parity, smoking status, passive smoking status, alcohol intake, number of hours spent outdoors, physical activity, folic acid intake, marital status, highest educational level, employment status, annual household income, type of residence, high-rise living, number of rooms in the house/apartment, materials covering the floor of the living room, age of house/apartment building, house renovation/interior completion after becoming pregnant, and number of years living in the current place of residence, with 19 regions set as a random effect.

Figure 2 shows the summary of the adjusted ORs (model 2) and their 95% Cis, which are presented in Table 2 and Table 3.

### 3.3. Results of Sensitivity Analysis

The results of the sensitivity analysis using the complete case dataset (Figure S1) were not meaningfully different from those calculated using the multiply imputed dataset.

The analysis using continuous scores of each developmental area in the ASQ-3 is summarized in Table S1.

## 4. Discussion

To our knowledge, this is the first study to examine the prospective relationships between the use of air purifiers and child neurodevelopment using a nationwide birth cohort study dataset (*n* > 80,000) [17,18,19] while controlling for up to 20 potential confounders. Analysis revealed that adjusted ORs (models 1 and 2) of air-purifier usage for screen-positive cases of developmental delay in the ASQ-3 were significantly lower than the reference (i.e., air-purifier nonuse) in the areas of fine motor and problem solving at 6 months and in communication, fine motor, problem solving, and personal–social at 12 months. In addition, adjusted ORs (models 1 and 2) at 12 months were lower than those at 6 months. Therefore, we found a negative relationship between the use of air purifiers during pregnancy and cases of neurodevelopmental delay that worsened over time until the child reached at least one year of age.

Although the usage of air purifiers was associated with the areas of communication, fine motor, problem solving, and personal–social, it is not clear which brain areas correspond to each developmental area in the ASQ-3. That said, the prefrontal cortex would be deeply involved in these areas because the prefrontal cortex is known to be responsible for higher intellectual functions, such as goal-driven behavior, including action planning and motor control, executive functioning, working memory, and adequate social response [28,29]. These functions are involved in the problem solving, communication, fine motor, and personal–social areas of the ASQ-3. This overlap suggests that air-purifier usage might be promote intact development in the prefrontal cortex. Further studies using magnetic resonance imaging and other developmental tests [7] will be interesting.

Given the underlying mechanism by which the usage of air purifiers prevents impaired neurodevelopment in infants via elimination of airborne PM [4,7,8,9,15], other methods that reduce PM would exert a similar effect in principle. Examples include wearing a mask, closing windows to shut out polluted air [30], ventilating a room [15,31], and temporarily residing in places with clean air. However, it should be noted that this working hypothesis regarding PM is one possibility among others. In fact, the factors associated with reduced risks for developmental delay through air-purifier usage are unknown. For example, other air pollutants, such as PAHs, can also mediate this relationship because prefrontal damage may be caused by PAH [8,32,33], whereas air purifiers can eliminate both vapor- and PM-phase PAHs [34]. Alternatively, developmental delay might be caused by something with no relation to air pollutants. Even in large-scale observational studies such as this one, it is virtually impossible to control all confounders and to come to a causal conclusion. Therefore, the observed relationships do not necessarily guarantee that the introduction of air purifiers will prevent infants from developing developmental delay. To examine the net effect of air-purifier usage on the prevention of developmental delay, it is necessary to conduct randomized controlled trials.

The crude ORs hardly changed even when 12 covariates were used (model 1) and all 20 covariates were used (model 2). The stability suggests that all covariates served only as very weak confounders. Thus, it would be very difficult to explain the observed association in terms of these variables, although air-purifier usage was clearly related to many variables, such as high education levels, high income, less smoking, being married, multipara, and living in a newer house (Table 1). Interestingly, these stable ORs were more salient at 12 months than at 6 months. This tendency implies that the common backgrounds, which could not be explained by the 20 covariates used, might emerge over time. Unfortunately, the JECS birth cohort data are currently available only until the first year of age, but data at later time points will be available in a few years. When the time is ripe, it will be fruitful to examine whether the differences between the use and nonuse of air purifiers increase or decrease over time.

Several limitations of this study should be addressed. First, we evaluated the usage of air purifiers using simple yes/no questions. Thus, important information regarding the performance of the devices, the types of air filters used, and their usage patterns (when, where, and how often the devices were used) was not available. In addition, we did not have data on the actual decrease in indoor PM levels after use of the air purifiers or on the dose–response relationship. Accordingly, the current estimation of ORs was rough. Second, the relationship between maternal PM2.5 exposure and subsequent developmental delay has not yet been reported for this population, so there is a gap in our hypothesis that air-purifier usage might prevent infant neurodevelopmental delay. We cannot deny the possibility that the observed relationship was a spurious association with the true cause existing outside the current framework. Third, although the ASQ-3 is a validated, parent-completed questionnaire, its aim is screening, not diagnosis. Thus, the developmental delay in this study could be overestimated. Fourth, our participants were recruited within the last 10 years from several areas across Japan, including both urban and rural areas, but the extent to which the results are applicable to specific populations cannot be determined. Finally, because our study used a prospective birth cohort, the observed prospective relationship cannot be regarded as a causal relationship.

Despite these limitations, we found inverse prospective relationships between the use of air purifiers during pregnancy and infant neurodevelopmental delay at 6 and 12 months postpartum that were salient over time in 82,441 mother–infant pairs while controlling for up to 20 potential confounders. Our results outline the potential role of air purifiers in inhibiting infant neurodevelopmental delay. This study provides a rationale for conducting randomized controlled trials to address the causal relationship between the use of air purifiers and infant neurodevelopmental delay and in-depth studies elucidating what factors are related to reduced risk for developmental delay in relation to air-purifier usage.

## Figures and Tables

**Table 1:** Characteristics of mothers and dwelling environments.

Variable	Category	Air-purifier Usage			
		Yes		No	
		*n* (%)		*n* (%)	
Overall		41,883	(50.8)	40,558	(49.2)
Mothers Age, years	<25	3661	(8.8)	4468	(11.1)
	25–<30	12,184	(29.2)	11,166	(27.6)
	30–<35	15,379	(36.9)	13,840	(34.3)
	≥35	10,500	(25.2)	10,937	(27.1)
Body mass index, kg/m^2^	<18.5	6748	(16.2)	6525	(16.2)
	18.5–<25	30,945	(74.2)	29,576	(73.2)
	≥25	4017	(9.6)	4287	(10.6)
Parity	Primipara	17,050	(40.9)	19,429	(48.1)
	Multipara	24,668	(59.1)	20,968	(51.9)
Smoking status	Never	24,257	(58.5)	23,708	(59.1)
	Former	15,687	(37.9)	14,586	(36.4)
	Current	1492	(3.6)	1797	(4.5)
Passive smoking status	Almost never	26,477	(63.6)	25,004	(62.0)
	Once a week	5109	(12.3)	4731	(11.7)
	2–3 times a week	3320	(8.0)	3341	(8.3)
	4–6 times a week	1906	(4.6)	2044	(5.1)
	Everyday	4818	(11.6)	5194	(12.9)
Alcohol intake	Never	13,816	(33.4)	13,455	(33.5)
	Former	26,532	(64.1)	25,492	(63.5)
	Current	1050	(2.5)	1195	(3.0)
Number of hours spent outdoors	<1	7310	(18.2)	7782	(20.1)
	1–<2	19,451	(48.4)	18,464	(47.7)
	2–<3	6579	(16.4)	5864	(15.1)
	≥3	6882	(17.1)	6631	(17.1)
Physical activity	No	8929	(22.1)	9334	(23.9)
	Yes	31,485	(77.9)	29,787	(76.1)
Quintile of folic acid intake, μg	≤151	7508	(18.0)	8486	(21.0)
	152–202	8303	(19.9)	8159	(20.2)
	203–256	8627	(20.7)	7982	(19.8)
	257–337	8641	(20.7)	7988	(19.8)
	≥338	8649	(20.7)	7792	(19.3)
Marital status	Married	40,131	(96.8)	37,838	(94.5)
	Single	1080	(2.6)	1786	(4.5)
	Divorced or widowed	233	(0.6)	409	(1.0)
Highest education level, years	≤12	13,469	(32.4)	15,275	(37.9)
	12–<16	18,555	(44.6)	16,286	(40.4)
	≥16	9554	(23.0)	8710	(21.6)
Employed	No	19,197	(46.3)	17,860	(44.6)
	Yes	22,272	(53.7)	22,214	(55.4)
Annual household income, million yen	<4	13,798	(35.3)	16,430	(43.8)
	4–<6	13,593	(34.8)	11,922	(31.8)
	≥6	11,697	(29.9)	9150	(24.4)
Dwelling environment type of residence					
	Wooden detached house	17,390	(41.9)	16,338	(40.6)
	Steel-frame detached house	2936	(7.1)	2240	(5.6)
	Wooden multiple dwelling house/apartment	4780	(11.5)	5285	(13.2)
	Steel-frame multiple dwelling house/apartment	16,032	(38.6)	15,943	(39.7)
	Other	390	(0.9)	393	(1.0)
High-rise living	No	39,609	(94.9)	38,766	(95.9)
	Yes	2122	(5.1)	1646	(4.1)
Number of rooms in the house/apartment	≤2	6990	(16.8)	8422	(20.9)
	3	13,212	(31.8)	13,240	(32.9)
	4	8559	(20.6)	7019	(17.5)
	5	6673	(16.1)	5602	(13.9)
	≥6	6096	(14.7)	5935	(14.8)
Materials covering the floor of the living room	Tatami (Japanese straw floor covering)	3893	(9.4)	5400	(13.4)
	Carpet on tatami	3237	(7.8)	4047	(10.1)
	Wooden flooring / tiles	15,606	(37.5)	13,470	(33.4)
	Carpet on wooden flooring/tiles	18,080	(43.5)	16,675	(41.4)
	Other	798	(1.9)	685	(1.7)
Age of house/apartment building, years	<1	2706	(6.5)	1943	(4.8)
	1–<3	5450	(13.1)	3792	(9.4)
	3–<5	4353	(10.5)	3426	(8.5)
	5–<10	6799	(16.4)	5885	(14.7)
	10–<20	9472	(22.8)	9575	(23.8)
	≥20	9433	(22.7)	11,071	(27.6)
	Unknown	3289	(7.9)	4480	(11.2)
House renovation/interior completion after becoming pregnant	No	40,092	(96.5)	39,035	(97.1)
	Yes	1435	(3.5)	1164	(2.9)
Number of years living in the current place of residence	<1	2753	(6.8)	2691	(6.9)
	1–<3	17,758	(43.8)	16,714	(42.7)
	3–<5	9581	(23.6)	8469	(21.6)
	5–<10	7121	(17.6)	7057	(18.0)
	10–<20	1936	(4.8)	2336	(6.0)
	≥20	1433	(3.5)	1868	(4.8)

**Table 2:** Prevalence, number, ORs and 95% CIs for the screen-detected cases of developmental delay in each area assessed using the ASQ-3 according to the use or nonuse of air purifiers.

	Prevalence	Cases	Total	Crude		Model 1		Model 2	
	%	*n*	*n*	OR	[95% CI]	OR	[95% CI]	OR	[95% CI]
6 months									
Communication									
AP use	1.90	797	41,883	0.97	[0.88, 1.08]	0.95	[0.85, 1.05]	0.95	[0.86, 1.06]
AP nonuse	1.95	791	40,558	Reference		Reference		Reference	
Gross motor									
AP use	1.75	734	41,883	0.92	[0.83, 1.03]	0.93	[0.83, 1.03]	0.92	[0.83, 1.03]
AP nonuse	1.92	778	40,558	Reference		Reference		Reference	
Fine motor									
AP use	2.58	1080	41,883	0.94	[0.86, 1.02]	**0.91**	**[0.83, 0.99]**	**0.91**	**[0.83, 0.99]**
AP nonuse	2.76	1120	40,558	Reference		Reference		Reference	
Problem solving									
AP use	3.48	1460	41,883	**0.83**	**[0.77, 0.89]**	**0.83**	**[0.77, 0.90]**	**0.83**	**[0.77, 0.90]**
AP nonuse	4.18	1694	40,558	Reference		Reference		Reference	
Personal–social									
AP use	1.43	598	41,883	0.97	[0.86, 1.09]	0.94	[0.83, 1.06]	0.95	[0.84, 1.07]
AP nonuse	1.48	598	40,558	Reference		Reference		Reference	
12 months									
Communication									
AP use	2.97	1245	41,883	**0.83**	**[0.77, 0.90]**	**0.86**	**[0.79, 0.93]**	**0.86**	**[0.79, 0.93]**
AP nonuse	3.50	1419	40,558	Reference		Reference		Reference	
Gross motor									
AP use	5.31	2222	41,883	0.95	[0.89, 1.01]	0.95	[0.89, 1.01]	0.95	[0.89, 1.01]
AP nonuse	5.59	2267	40,558	Reference		Reference		Reference	
Fine motor									
AP use	5.13	2148	41,883	**0.86**	**[0.81, 0.91]**	**0.87**	**[0.81, 0.92]**	**0.87**	**[0.82, 0.92]**
AP nonuse	5.96	2418	40,558	Reference		Reference		Reference	
Problem solving									
AP use	4.49	1879	41,883	**0.78**	**[0.74, 0.84]**	**0.82**	**[0.77, 0.88]**	**0.83**	**[0.77, 0.88]**
AP nonuse	5.65	2291	40,558	Reference		Reference		Reference	
Personal–social									
AP use	2.65	1108	41,883	**0.80**	**[0.73, 0.87]**	**0.79**	**[0.72, 0.86]**	**0.79**	**[0.72, 0.86]**
AP nonuse	3.27	1326	40,558	Reference		Reference		Reference	

OR, odds ratio; CI, confidence interval; AP, air purifier; ASQ-3, Ages & Stages Questionnaire, Third Edition. Based on imputed data for the 82,441 infants in this study. Boldface indicates statistical significance at the level of 5%. Crude = crude model only, with 19 regions set as a random effect. Model 1 = partial model adjusted for maternal age, parity, smoking status, passive smoking status, folic acid intake, marital status, highest educational level, annual household income, type of residence, number of rooms in the house/apartment, materials covering the floor of the living room, and age of house/apartment building, with the region set as a random effect. Model 2 = full model adjusted for all the covariates in model 1: body mass index, alcohol intake, number of hours spent outdoors, physical activity, employment status, high-rise living, house renovation/interior completion after becoming pregnant, and number of years living in the current place of residence.

**Table 3:** Total numbers of screen-positive cases over the five developmental areas in the ASQ-3 and their ORs according to the use or nonuse of air purifiers.

	Mean	SD	Crude		Model 1		Model 2	
			OR	[95% CI]	OR	[95% CI]	OR	[95% CI]
6 months								
AP use	0.11 ±	0.43	**0.91**	**[0.87, 0.95]**	**0.90**	**[0.86, 0.94]**	**0.90**	**[0.86, 0.94]**
AP nonuse	0.12 ±	0.45	Reference		Reference		Reference	
12 months								
AP use	0.21 ±	0.60	**0.85**	**[0.82, 0.87]**	**0.86**	**[0.84, 0.89]**	**0.86**	**[0.84, 0.89]**
AP nonuse	0.24 ±	0.65	Reference		Reference		Reference	

OR, odds ratio; CI, confidence interval; AP, air purifier; ASQ-3, Ages & Stages Questionnaire, Third Edition. Based on imputed data for the 82,441 infants in this study. Boldface indicates statistical significance at the level of 5%. Crude = crude model only, with 19 regions set as a random effect. Model 1 = partial model adjusted for maternal age, parity, smoking status, passive smoking status, folic acid intake, marital status, highest educational level, annual household income, type of residence, number of rooms in the house/apartment, materials covering the floor of the living room, and age of house/apartment building, with the region set as a random effect. Model 2 = full model adjusted for all the covariates in model 1: body mass index, alcohol intake, number of hours spent outdoors, physical activity, employment status, high-rise living, house renovation/interior completion after becoming pregnant, and number of years living in the current place of residence.

## Data Availability

The data used to derive our conclusions are unsuitable for public deposition because of ethical restrictions and the specific legal framework in Japan. Furthermore, the Ethical Guidelines for Epidemiological Research enforced by the Japanese Ministry of Education, Culture, Sports, Science and Technology and the Ministry of Health, Labour and Welfare restrict the open sharing of epidemiological data. All inquiries about access to data should be sent to: jecs-en@nies.go.jp. The person responsible for handling enquiries sent to this e-mail address is Dr. Shoji F. Nakayama, JECS Programme Office, National Institute for Environmental Studies.

## Supplementary Materials

The following are available online at https://www.mdpi.com/2077-0383/9/6/1924/s1, Figure S1: Adjusted ORs (model 2) and 95% confidence intervals (bars) of air-purifier usage for screen-positive cases of developmental delay assessed using the ASQ-3 with air-purifier nonuse set as a reference, Table S1: Mean and SD values, βs and 95% CIs for the score of each developmental area in the ASQ-3 according to the use or nonuse of air purifiers, SAS code for main analysis.

## References

[1] Landrigan P.J., Fuller R., Acosta N.J.R., Adeyi O., Arnold R., Basu N., Balde A.B., Bertollini R., Bose-O’Reilly S., Boufford J.I. (2018). The Lancet Commission on pollution and health. Lancet.

[2] Forouzanfar M.H., Afshin A., Alexander L.T., Anderson H.R., Bhutta Z.A., Biryukov S., Brauer M., Burnett R., Cercy K., Charlson F.J. (2016). Global, regional, and national comparative risk assessment of 79 behavioural, environmental and occupational, and metabolic risks or clusters of risks, 1990–2015: A systematic analysis for the Global Burden of Disease Study 2015. Lancet.

[3] Landrigan P.J. (2017). Air pollution and health. Lancet Public Health.

[4] Suades-Gonzalez E., Gascon M., Guxens M., Sunyer J. (2015). Air pollution and neuropsychological development: A review of the latest evidence. Endocrinology.

[5] Brockmeyer S., D’Angiulli A. (2016). How air pollution alters brain development: The role of neuroinflammation. Transl. Neurosci..

[6] Morawska L., Ayoko G.A., Bae G.N., Buonanno G., Chao C.Y.H., Clifford S., Fu S.C., Hanninen O., He C., Isaxon C. (2017). Airborne particles in indoor environment of homes, schools, offices and aged care facilities: The main routes of exposure. Environ. Int..

[7] Guxens M., Lubczynska M.J., Muetzel R.L., Dalmau-Bueno A., Jaddoe V.W.V., Hoek G., Van der Lugt A., Verhulst F.C., White T., Brunekreef B. (2018). Air pollution exposure during fetal life, brain morphology, and cognitive function in school-age children. Biol. Psychiatry.

[8] Perera F.P., Li Z.G., Whyatt R., Hoepner L., Wang S.A., Camann D., Rauh V. (2009). Prenatal airborne polycyclic aromatic hydrocarbon exposure and child IQ at age 5 years. Pediatrics.

[9] Guxens M., Garcia-Esteban R., Giorgis-Allemand L., Forns J., Badaloni C., Ballester F., Beelen R., Cesaroni G., Chatzi L., de Agostini M. (2014). Air pollution during pregnancy and childhood cognitive and psychomotor development: Six European birth cohorts. Epidemiology.

[10] Rice D., Barone S. (2000). Critical periods of vulnerability for the developing nervous system: Evidence from humans and animal models. Environ. Health Perspect..

[11] Grandjean P., Landrigan P.J. (2014). Neurobehavioural effects of developmental toxicity. Lancet Neurol..

[12] Calderon-Garciduenas L., Mora-Tiscareno A., Ontiveros E., Gomez-Garza G., Barragan-Mejia G., Broadway J., Chapman S., Valencia-Salazar G., Jewells V., Maronpot R.R. (2008). Air pollution, cognitive deficits and brain abnormalities: A pilot study with children and dogs. Brain Cogn..

[13] Maher B.A., Ahmed I.A., Karloukovski V., MacLaren D.A., Foulds P.G., Allsop D., Mann D.M., Torres-Jardon R., Calderon-Garciduenas L. (2016). Magnetite pollution nanoparticles in the human brain. Proc. Natl. Acad. Sci. USA.

[14] Fermo P., Comite V., Falciola L., Guglielmi V., Miani A. (2019). Efficiency of an Air Cleaner Device in Reducing Aerosol Particulate Matter (PM) in Indoor Environments. Int. J. Environ. Res. Public Health.

[15] Kanatani K.T., Okumura M., Tohno S., Adachi Y., Sato K., Nakayama T. (2014). Indoor particle counts during Asian dust events under everyday conditions at an apartment in Japan. Environ. Health Prev. Med..

[16] Jia-Ying L., Zhao C., Jia-Jun G., Zi-Jun G., Xiao L., Bao-Qing S. (2018). Efficacy of air purifier therapy in allergic rhinitis. Asian Pac. J. Allergy Immunol..

[17] Kawamoto T., Nitta H., Murata K., Toda E., Tsukamoto N., Hasegawa M., Yamagata Z., Kayama F., Kishi R., Ohya Y. (2014). Rationale and study design of the Japan environment and children’s study (JECS). BMC Public Health.

[18] Michikawa T., Nitta H., Nakayama S.F., Yamazaki S., Isobe T., Tamura K., Suda E., Ono M., Yonemoto J., Iwai-Shimada M. (2018). Baseline profile of participants in the Japan Environment and Children’s Study (JECS). J. Epidemiol..

[19] Iwai-Shimada M., Nakayama S.F., Isobe T., Michikawa T., Yamazaki S., Nitta H., Takeuchi A., Kobayashi Y., Tamura K., Suda E. (2018). Questionnaire results on exposure characteristics of pregnant women participating in the Japan Environment and Children Study (JECS). Environ. Health Prev. Med..

[20] Squires J., Bricker D. (2009). Ages & Stages Questionnaires (ASQ-3): A Parent-Completed Child-Monitoring System.

[21] Korfmacher J., Chawla N. Toolkit of Recommended Curricula and Assessments for Early Childhood Home Visiting. Geneva: UNICEF. https://www.unicef.org/eca/sites/unicef.org.eca/files/2017-11/Toolkit_of_Recommended_Curricula_and_Assessments_for_Home_Visiting_0.pdf.

[22] Gao Y., Sheng C., Xie R.H., Sun W., Asztalos E., Moddemann D., Zwaigenbaum L., Walker M., Wen S.W. (2016). New perspective on impact of folic acid supplementation during pregnancy on neurodevelopment/autism in the offspring children—A systematic review. PLoS ONE.

[23] Fujiwara T., Michikawa T., Suzuki K., Takebayashi T., Yamagata Z. (2014). Impact of high-rise living on children’s development and health: A critical review of literature. Yamanashi Med. J..

[24] Hamazaki K., Takamori A., Tsuchida A., Kigawa M., Tanaka T., Ito M., Adachi Y., Saito S., Origasa H., Inadera H. (2018). Dietary intake of fish and n-3 polyunsaturated fatty acids and risks of perinatal depression: The Japan Environment and Children’s Study (JECS). J. Psychiatr. Res..

[25] Matsumura K., Hamazaki K., Tsuchida A., Kasamatsu H., Inadera H. (2019). Education level and risk of postpartum depression: Results from the Japan Environment and Children’s Study (JECS). BMC Psychiatry.

[26] Van Buuren S. (2007). Multiple imputation of discrete and continuous data by fully conditional specification. Stat. Methods Med Res..

[27] Rubin D.B. (2004). Multiple Imputation for Nonresponse in Surveys.

[28] Guyton A.C., Hall J.E. (1996). Human Physiology and Mechanisms of Disease.

[29] Miller E.K., Cohen J.D. (2001). An integrative theory of prefrontal cortex function. Annu. Rev. Neurosci..

[30] Itazawa T., Kanatani K.T., Hamazaki K., Inadera H., Tsuchida A., Tanaka T., Nakayama T., Go T., Onishi K., Kurozawa Y. (2020). The impact of exposure to desert dust on infants’ symptoms and countermeasures to reduce the effects. Allergy.

[31] Vrijheid M., Martinez D., Aguilera I., Bustamante M., Ballester F., Estarlich M., Fernandez-Somoano A., Guxens M., Lertxundi N., Martinez M.D. (2012). Indoor air pollution from gas cooking and infant neurodevelopment. Epidemiology.

[32] Perera F.P., Rauh V., Tsai W.Y., Kinney P., Camann D., Barr D., Bernert T., Garfinkel R., Tu Y.H., Diaz D. (2003). Effects of transplacental exposure to environmental pollutants on birth outcomes in a multiethnic population. Environ. Health Perspect..

[33] Peterson B.S., Rauh V.A., Bansal R., Hao X., Toth Z., Nati G., Walsh K., Miller R.L., Arias F., Semanek D. (2015). Effects of prenatal exposure to air pollutants (polycyclic aromatic hydrocarbons) on the development of brain white matter, cognition, and behavior in later childhood. JAMA Psychiatry.

[34] Ma Y., Harrad S. (2015). Spatiotemporal analysis and human exposure assessment on polycyclic aromatic hydrocarbons in indoor air, settled house dust, and diet: A review. Environ. Int..

