# Supplementary material for: Prospective Association of Air-Purifier Usage during Pregnancy with Infant Neurodevelopment: A Nationwide Longitudinal Study—Japan Environment and Children’s Study (JECS)
Source: J Clin Med. 2020 Jun 19;9(6):1924. doi: 10.3390/jcm9061924 (PMC7356334; doi:10.3390/jcm9061924)
Supplement: Supplementary file 1 [file jcm-09-01924-s001.pdf]

Supplementary material

Prospective association of air-purifier usage during pregnancy with infant neurodevelopment: A nationwide longitudinal study – Japan Environment and Children's Study (JECS)

Kenta Matsumura [1,*], Kei Hamazaki [1,2], Akiko Tsuchida [1,2], Hidekuni Inadera [1,2] and the Japan Environment and Children's Study (JECS) Group [3]

[1] Toyama Regional Center for Japan Environment and Children's Study, University of Toyama, 2630 Sugitani, Toyama, Toyama 930-0194, Japan; kmatsumu@med.u-toyama.ac.jp (K.M.); keihama@med.u-toyama.ac.jp (K.H.); aktsuchi@med.u-toyama.ac.jp (A.T.); inadera@med.u-toyama.ac.jp (H.I.)

[2] Department of Public Health, Faculty of Medicine, University of Toyama, 2630 Sugitani, Toyama, Toyama 930-0194, Japan

[3] Japan Environment and Children's Study Programme Office, National Institute for Environmental Studies, 16-2 Onogawa, Tsukuba, Ibaraki 305-8506, Japan; jecscore@nies.go.jp

* Correspondence: kmatsumu@med.u-toyama.ac.jp; Tel.: +81-76-434-7277

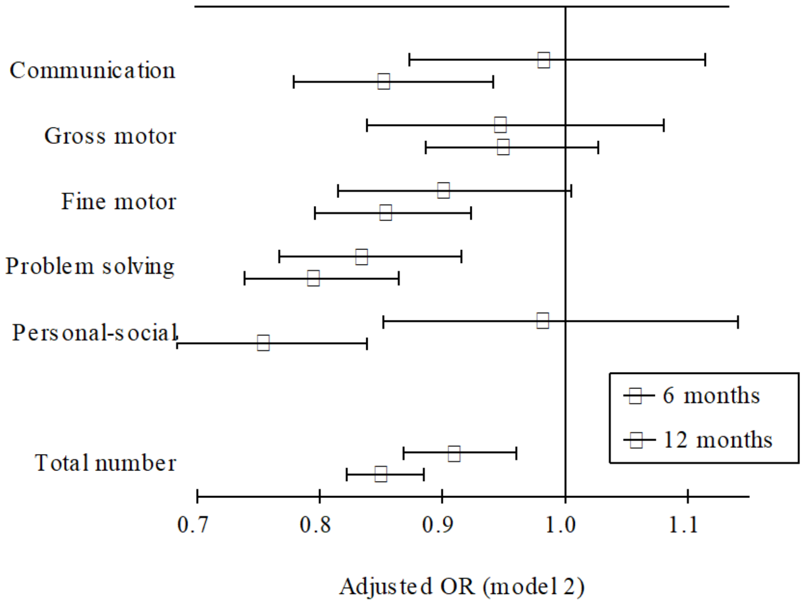

Figure S1. Adjusted ORs (model 2) and 95% confidence intervals (bars) of air-purifier usage for screen-positive cases of developmental delay assessed using the ASQ-3 with air-purifier nonuse set as a reference.

This graph was drawn using the results of the complete case analysis.

OR, odds ratio; ASQ-3, Ages & Stages Questionnaire, Third edition; Total number, total number of screen-positive cases over five developmental areas in the ASQ-3.

Model 2 = full model adjusted for maternal age, body mass index, parity, smoking status, passive smoking status, alcohol intake, number of hours spent outdoors, physical activity, folic acid intake, marital status, highest educational level, employment status, annual household income, type of residence, high-rise living, number of rooms in the house/apartment, living-room flooring material, age of house/apartment building, house renovation/interior completion after becoming pregnant, and number of years living in the current place of residence, with 19 regions set as a random effect.

Table S1. Mean and SD values, βs and 95% CIs for the score of each developmental area in the ASQ-3 according to the use or nonuse of air purifiers.

	M		SD	Crude		Model1		Model2	
				β	[95% CI]	β	[95% CI]	β	[95% CI]
6 months									
Communication									
AP use	46.6	±	9.1	-0.06	[-0.19, 0.06]	0.10	[-0.02, 0.23]	0.10	[-0.03, 0.22]
AP nonuse	46.7	±	9.1	Reference		Reference		Reference	
Gross motor									
AP use	33.8	±	13.0	**0.37**	**[0.19, 0.55]**	**0.32**	**[0.14, 0.50]**	**0.32**	**[0.14, 0.50]**
AP nonuse	33.4	±	13.2	Reference		Reference		Reference	
Fine motor									
AP use	41.1	±	14.4	**0.36**	**[0.16, 0.56]**	**0.34**	**[0.15, 0.54]**	**0.32**	**[0.12, 0.52]**
AP nonuse	40.7	±	14.5	Reference		Reference		Reference	
Problem solving									
AP use	44.5	±	12.7	**0.49**	**[0.31, 0.67]**	**0.54**	**[0.36, 0.72]**	**0.53**	**[0.35, 0.71]**
AP nonuse	43.9	±	13.2	Reference		Reference		Reference	
Personal-social									
AP use	34.7	±	15.5	**0.62**	**[0.41, 0.84]**	**0.70**	**[0.48, 0.91]**	**0.68**	**[0.46, 0.90]**
AP nonuse	34.1	±	15.7	Reference		Reference		Reference	
12 months									
Communication									
AP use	38.4	±	13.8	**1.10**	**[0.91, 1.28]**	**1.04**	**[0.85, 1.23]**	**1.03**	**[0.84, 1.22]**
AP nonuse	37.4	±	14.0	Reference		Reference		Reference	
Gross motor									
AP use	43.4	±	18.6	**0.76**	**[0.51, 1.01]**	**0.68**	**[0.43, 0.93]**	**0.67**	**[0.42, 0.92]**
AP nonuse	42.6	±	18.4	Reference		Reference		Reference	
Fine motor									
AP use	48.8	±	11.8	**0.76**	**[0.59, 0.93]**	**0.71**	**[0.54, 0.88]**	**0.70**	**[0.53, 0.87]**
AP nonuse	48.0	±	12.4	Reference		Reference		Reference	
Problem solving									
AP use	43.1	±	14.0	**1.33**	**[1.13, 1.52]**	**0.93**	**[0.73, 1.12]**	**0.91**	**[0.71, 1.10]**
AP nonuse	41.8	±	14.6	Reference		Reference		Reference	
Personal-social									
AP use	37.8	±	15.2	**1.04**	**[0.84, 1.25]**	**1.06**	**[0.85, 1.27]**	**1.05**	**[0.84, 1.25]**
AP nonuse	36.8	±	15.3	Reference		Reference		Reference	

β, delta difference; CI, confidence interval; AP, air purifier; ASQ-3, Ages & Stages Questionnaire, Third edition

Based on imputed data for the 82,441 infants in this study.

Boldface indicates statistical significance at the level of 5%.

The total score for each area ranges from 0 to 60 points (the higher, the better).

Crude = crude model only, with 19 regions set as a random effect.

Model 1 = partial model adjusted for maternal age, parity, smoking status, passive smoking status, folic acid intake, marital status, highest educational level, annual household income, type of residence, number of rooms in the house/apartment, materials covering the floor of the living room, and age of house/apartment building, with the region set as a random effect.

Model 2 = full model adjusted for all the covariates in model 1: body mass index, alcohol intake, number of hours spent outdoors, physical activity, employment status, high-rise living, house renovation/interior completion after becoming pregnant, and number of years living in the current place of residence.

SAS code for multiple imputation (MI) and generalized linear mixed models (GLMMs) for MI.

/* MI */

```
proc mi nimpute=24 data=ageof01_196_all seed=6342 out=outmi;
class       mt1_marrige_status
            mt2_house_type
            unit_rc_no
            c_sex
            baby_anomaly
            mt2_work_ver2
            mt2_smoking_ver2
            mt2_p_smo_freq
            mt2_activity_ver2
            mt2_alcohol_ver2
            mt2_income_ver2
            mt2_school_ver2
            mt2_school_hus_ver2
            DrT1_parity_ver2
            dr0m_preg_compli
            mt2_house_age
            mt2_ext_carpe
            mt2_living_floor
            mt2_air_purifi
            m1m_lactate
;
fcs reg (    m_height
            m_weightb
            MT2_m_age
            mt2_energy
            mt2_fish
            mt2_folicA_mg
            mt2_income_n
            birth_weeks
            MT1_metsmin_av_m
            MT2_metsmin_av_m
            mt2_outdoor_time_2
            mt2_live_year
            mt2_rooms
            C6m_ASQ_aComu C6m_ASQ_bGM C6m_ASQ_cFM C6m_ASQ_dProblem C6m_ASQ_ePersonal
            C1y_ASQ_aComu C1y_ASQ_bGM C1y_ASQ_cFM C1y_ASQ_dProblem C1y_ASQ_ePersonal
)

discrim (   mt1_marrige_status
            mt2_house_type
            unit_rc_no
            c_sex
            baby_anomaly
            mt2_smoking_ver2
            mt2_activity_ver2
            mt2_alcohol_ver2
            mt2_ext_carpe
            dr0m_preg_compli
            mt2_air_purifi
            mt2_living_floor
            / classeffects=include
)
```

```
logistic (  m1m_lactate
            mt2_work_ver2
            mt2_p_smo_freq
            mt2_income_ver2
            mt2_school_ver2
            mt2_school_hus_ver2
            mt2_house_age
            DrT1_parity_ver2
            / LIKELIHOOD=AUGMENT
);

var

/* continuous*/
            m_height
            m_weightb
            MT2_m_age
            mt2_energy
            mt2_fish
            mt2_folicA_mg
            mt2_income_n
            birth_weeks
            MT1_metsmin_av_m
            MT2_metsmin_av_m
            mt2_outdoor_time_2
            mt2_live_year
            mt2_rooms
            C6m_ASQ_aComu C6m_ASQ_bGM C6m_ASQ_cFM C6m_ASQ_dProblem C6m_ASQ_ePersonal
            C1y_ASQ_aComu C1y_ASQ_bGM C1y_ASQ_cFM C1y_ASQ_dProblem C1y_ASQ_ePersonal

/* categorical */
            mt1_marrige_status
            mt2_house_type
            unit_rc_no
            c_sex
            baby_anomaly
            mt2_smoking_ver2
            mt2_activity_ver2
            mt2_alcohol_ver2
            dr0m_preg_compli
            mt2_ext_carpe
            mt2_living_floor
            mt2_air_purifi
            m1m_lactate

/*order*/
            mt2_work_ver2
            mt2_p_smo_freq
            mt2_school_ver2
            mt2_school_hus_ver2
            mt2_income_ver2
            mt2_house_age
            DrT1_parity_ver2
;
run;
```

/* GLMMs for MI */

```
/* 1st part: GLMMs for each imputed dataset*/

ods select none;
proc glimmix data=outmi method=quad;
class mt2_house_type(ref="1");
class mt2_house_age(ref="1");
class mt2_live_year_ver2(ref="0");
class mt2_kousou(ref="0");
class mt2_rooms_ver2(ref="2");
class mt2_ext_carpe(ref="2");

class mt2_air_purifi(ref="2");

class mt2_living_floor(ref="1");
class mt2_income_ver2(ref="1");
class mt2_school_ver2(ref="1");
class mt1_marrige_status(ref="1");
class mt1_BMI_ver2(ref="2");
class mt2_smoking_ver2(ref="1");
class mt2_p_smo_freq(ref="1");
class mt2_activity_ver2(ref="0");
class mt2_alcohol_ver2(ref="1");
class drt1_parity_ver2(ref="0");
class mt2_work_ver2(ref="0");
class mt2_m_age_ver2(ref="1");
class qn_mt2_folicA_mg(ref="0");
class mt2_out_t_ver2(ref="0");
class unit_rc_no;

model c6m_comm_case =
/*model c6m_gm_case =*/
/*model c6m_fm_case =*/
/*model c6m_prob_case =*/
/*model c6m_pers_case =*/
/*model c6m_asq_total/N_asq=*/            /* N_asq = 5;*/

/*model c1y_gm_case =*/
/*model c1y_fm_case =*/
/*model c1y_prob_case =*/
/*model c1y_pers_case =*/
/*model c1y_asq_total/N_asq=*/

        mt2_house_type
        mt2_house_age
        mt2_live_year_ver2
        mt2_kousou
        mt2_rooms_ver2
        mt2_ext_carpe

        mt2_air_purifi

        mt2_living_floor
        mt2_income_ver2
        mt2_school_ver2
        mt1_marrige_status
```

```
        mt1_BMI_ver2
        mt2_smoking_ver2
        mt2_p_smo_freq
        mt2_activity_ver2
        mt2_alcohol_ver2/
        drt1_parity_ver2
        mt2_work_ver2
        MT2_m_age_ver2

        qn_mt2_folicA_mg
        mt2_out_t_ver2

        / dist = binomial solution covb oddsratio ddfm = bw;
        random intercept / subject = unit_rc_no;

by _Imputation_;
ods output ParameterEstimates=gparms;
run;
ods select all;


/* 2nd part: combining estimates using Rubin's rule */

proc mianalyze parms=gparms;
class mt2_house_type;
class mt2_house_age;
class mt2_live_year_ver2;
class mt2_kousou;
class mt2_rooms_ver2;
class mt2_ext_carpe;

class mt2_air_purifi;

class mt2_living_floor;
class mt2_income_ver2;
class mt2_school_ver2;
class mt1_marrige_status;
class mt1_BMI_ver2;
class mt2_smoking_ver2;
class mt2_p_smo_freq;
class mt2_activity_ver2;
class mt2_alcohol_ver2;
class drt1_parity_ver2;
class mt2_work_ver2;
class mt2_m_age_ver2;
class qn_mt2_folicA_mg;
class mt2_out_t_ver2;

modeleffects Intercept

        mt2_house_type
        mt2_house_age
        mt2_live_year_ver2
        mt2_kousou
        mt2_rooms_ver2
        mt2_ext_carpe
```

```
            mt2_air_purifi

            mt2_living_floor
            mt2_income_ver2
            mt2_school_ver2
            mt1_marrige_status
            mt1_BMI_ver2
            mt2_smoking_ver2
            mt2_p_smo_freq
            mt2_activity_ver2
            mt2_alcohol_ver2
            drt1_parity_ver2
            mt2_work_ver2
            MT2_m_age_ver2
            qn_mt2_folicA_mg
            mt2_out_t_ver2
            ;
run;
```
